# Religion in Medical Addresses Endorsed; the Minority Report

**Published:** 1886-08

**Authors:** 


### RELIGION IN MEDICAL ADDRESSES ENDORSED.

### THE MINORITY REPORT.

CUERO, DeWitt Co., Texas, July 30, 1886.

DR. F. E. DANIEL, AUSTIN, TEXAS.

DEAR SIR :

I return to your address by this mail No. 1, Vol. II DANIEL'S MEDICAL JOURNAL. As my subscription is paid to date you will erase my name from your list. I *fully* support Dr. E. P. Beckton and do not support your editorial of above issue·

Yours &c.

D. B. BLAKE.

Here now is the legitimate fruit of our temerity in saying the profession of Texas do not approve of the introduction of theology into the Presidential address before the State Medical Association ! We have lost a subscriber ! He stops his Journal because he endorses the plan of mixing up religion in the matters of the State Medical Association a d of denouncing *science*, as damning to the soul and withering to the body. We submit, we have not questioned the orthodoxy of Dr Becton's religious belief, nor have we said *we do not* endorse his religious views *if expressed in the pulpit.* We have protested that the Medical profession of Texas do not agree with Dr. Becton that the teachings of " such men as Darwin and Huxley" are ruinous—to mind, body, soul and estate, and do not sustain him in his denounciation of them and their labors. If, perchance, Dr. Blake has been tampering with science and has found Dr. Benton's statements true as regards himself, we can only say his is an isolated case, and we trust he may not be "frozen," "damned," "withered" and "blighted" past all hope of redemption.

